# Oncology and Acute Care Surgery: Examining Outcomes in Cancer Patients Requiring Emergent Laparotomy

**DOI:** 10.1016/j.jss.2025.03.015

**Published:** 2025-04-23

**Authors:** Grace M. Mallampalli, Drayson B. Campbell, Shruthi Srinivas, Holly Baselice, Courtney M. Collins, Megan Mansour, Jordan M. Cloyd, Jonathan Wisler

**Affiliations:** a The Ohio State University College of Medicine, Columbus, Ohio; b Department of Surgery, The Ohio State University Wexner Medical Center, Columbus, Ohio

**Keywords:** Abdominal surgery, Active cancer, Cancer outcomes, Complications, Emergency surgery, Emergent laparotomy, Palliative care

## Abstract

**Introduction:**

Patients presenting with abdominal emergencies requiring emergent laparotomy are at high risk of morbidity and mortality. The impact of an active cancer diagnosis on the short-term outcomes of emergent laparotomy is not well described.

**Methods:**

A retrospective analysis was conducted using an institutional database comprised of patients admitted from 2015 to 2019 who underwent exploratory laparotomy within 6 h of surgical consultation. The postoperative outcomes of patients with and without an active cancer diagnosis were compared using univariate and multivariable analysis with adjustment for clinical and demographic variables.

**Results:**

Among 409 patients who met inclusion criteria, 320 (78.2%) were without an active cancer diagnosis and 89 (21.8%) had an active cancer diagnosis. Patients with cancer were older (median [interquartile range], 63 [56, 70] y *versus* 59 y [49, 69], *P* = 0.0001) and presented with higher number of acute severity measures (17.9% *versus* 13.1%, *P* = 0.017). Patients with cancer had higher odds of 90-d mortality (adjusted odds ratio: 12.0, 95% confidence interval: [4.9, 29.3]) and 90-d systemic complications (adjusted odds ratio 2.4, 95% confidence interval: [1.26, 4.50]). Patients with cancer who died during index hospitalization received more inpatient palliative care consultations (53.3% *versus* 25.7%, *P* = 0.0336) and experienced more systemic complications postoperatively (45.2% *versus* 17.0%, *P* = 0.0038).

**Conclusions:**

An active cancer diagnosis is independently associated with worse postoperative outcomes following emergent laparotomy. These findings should inform individual and shared decision-making regarding the role of emergency surgery and are relevant for future patient-centered research in oncologic emergencies.

## Introduction

Patients presenting with abdominal emergencies requiring emergent laparotomy are at high risk for morbidity and mortality. Emergency general surgery (EGS) patients are the most at-risk surgical patients, experiencing disproportionate rates of complications and mortality compared to non-EGS patients. This disparity is especially true in patients requiring open operative approach. EGS patients undergoing open gastrointestinal surgery are at increased risk of 30-d mortality compared with patients undergoing elective open gastrointestinal surgery.^1^

Abdominal pathologies often requiring emergent surgical intervention include closed-loop intestinal obstruction, perforated viscus, septic complications (e.g., peritonitis), and hemorrhage. An important patient population susceptible to abdominal emergencies includes patients with a cancer diagnosis. Abdominal emergencies account for over 40% of emergency department visits among oncologic patients and often require emergency surgery.^2^ Abdominal emergencies in the oncologic patient are often a result of disease progression, tumor burden, systemic manifestations such as paraneoplastic syndrome, or cancer treatment.^3^

Oncologic patients are known to be at greater risk for adverse surgical outcomes relative to patients without known malignancy.^4,5^ Previous literature has shown that active malignancy and emergency abdominal surgery are independently poor prognostic factors.^6,7^ However, limited research has been conducted to investigate the impact of active cancer on outcomes following emergent laparotomy for acute abdominal pathology. The existing literature demonstrates that surgeons are generally able to predict emergent surgery outcomes in advanced cancer patients to better identify those who could benefit from perioperative palliative care, but there is a gap in research pertaining specifically to the relationship between cancer and emergent laparotomy outcomes.^6^

Our primary objective is to compare mortality of patients with active cancer with that of patients without active cancer. Secondary outcomes include systemic complications within 90 d of laparotomy and discharge disposition. We hypothesize that patients with cancer will experience a higher rate of mortality and will be discharged home to care for themselves less frequently than patients without cancer.

## Methods

### Study overview

A retrospective chart review was performed of all patients cared for at a large academic tertiary hospital from October 1, 2015, to December 31, 2019, who underwent an exploratory laparotomy by an EGS service within 6 hs of initial surgical consultation. Patients were excluded if they underwent laparotomy by a non-EGS service or for trauma. Patients were also excluded if they had a new diagnosis of cancer found on laparotomy for emergent pathology or if they had previously undergone oncologic treatment, other than surgery alone, but were no longer receiving radiation or systemic therapy. The cohort was divided into the following two subgroups: patients with no cancer and patients with active cancer. Active cancer was defined as currently receiving oncologic treatment (chemotherapy, radiation, immunotherapy, hormone therapy, etc.) or undergoing workup for new cancer diagnosis. Patients with prior oncologic diagnosis were designated to the “no cancer” cohort, including those undergoing surveillance for their previous oncologic diagnosis. Data were analyzed at the cohort and subgroup level for comparative purposes. The study was approved by our center’s institutional review board (IRB#2021H0077). Waiver of consent was granted by the institutional review board.

### Data collection

Data were collected on patient demographics, including sex, age, ethnicity, race, insurance type, employment status, and ability to live independently. Social work data on quality of social support were also queried. Based on social work documentation of living situation, available support, and quality of support system, patients’ social support was subjectively categorized into “unsupportive”, “supportive”, and “very supportive”. In terms of clinical characteristics, data were gathered on patient presenting location, including home or transfer from another facility such as a skilled nursing facility, rehabilitation center, or other hospital. Data were also gathered on Elixhauser comorbidities. Acuity of presentation was assessed in terms of number of acute severity measures (ASMs), with a larger number indicating a more severe presentation. ASMs assessed included need for dialysis, new psychosis, cardiac arrest, coma, ventilator dependence, pneumonia, ascites, open abdominal wound, use of stress dose steroids, medication-induced coagulopathy, sepsis, and recent transfusion. Preoperative diagnosis was localized into five domains, (1) foregut (esophagus and stomach); (2) small bowel; (3) large bowel and/or rectum; (4) nonenteric organ such as gallbladder, appendix, pancreas, or liver; and (5) unable to determine. Preoperative diagnosis was designated based on initial surgical diagnosis requiring laparotomy and confirmed with intraoperative findings. Discharge disposition was recorded. Systemic complications (shock, cardiac, pulmonary, neurological, genitourinary, and thromboembolic) and mortality were captured at discharge and at 90 d postdischarge.

Study data were collected and managed using Research Electronic Data Capture electronic data capture tools hosted at the study site. Research Electronic Data Capture is a secure, web-based software platform designed to support data capture for research studies, providing (1) an intuitive interface for validated data capture; (2) audit trails for tracking data manipulation and export procedures; (3) automated export procedures for seamless data downloads to common statistical packages; and (4) procedures for data integration and interoperability with external sources.^8,9^

### Cancer characteristics

Specific data were then obtained on the subgroup of patients with active cancer. Cancer was stratified by subtype, (1) foregut, (2) colorectal/anal, (3) hepatopancreatobiliary, (4) breast/skin/soft tissue, (5) blood, and 6) other. Data were obtained on whether patients had stage IV disease; whether they were on active treatment with chemotherapy, radiation, immunotherapy, or hormonal therapy; and whether they had progressed or responded to the treatment at that time. Data were also gathered on palliative care consultation for patients with active malignancies. Further subgroups were then generated within the active cancer subgroup to compare admission mortality and systemic complications.

### Statistical analysis

Subgroups were described through summary statistics, medians, and interquartile ranges (IQRs) for continuous data, and sums and proportions for categorical data. Chi-squared tests and Student’s *t*-tests were performed for statistical significance between the active cancer and no active cancer subgroups. A *P* value of ≤0.05 was considered significant. Adjusted odds ratios (aORs) were calculated to compare the cancer and non-cancer subgroups, adjusting for age, sex, race, ASMs, and Elixhauser comorbidities. To compare systemic complications and mortality based on cancer subtype and clinical management including chemotherapy, radiation, and disease progression, aORs were further calculated, adjusting for age, sex, race, ASM, and Elixhauser comorbidities, and metastatic cancer. The Strengthening the Reporting of Observational Studies in Epidemiology (STROBE) guideline was used to ensure proper reporting of methods, results, and discussion. All statistics were computed with Statistical Analysis System (SAS; version 9.4, SAS Software, Cary, NC).

## Results

### Demographics

Of the 409 patients who underwent emergency laparotomy, 89 (21.8%) had active cancer (Table 1). Patients with active cancer were older than patients without active cancer (63 y [56, 70] *versus* 59 y [49, 69], median [IQR], *P* = 0.0001). However, no differences were observed when compared by sex, ethnicity, race, insurance status, employment status, living status, or quality of social support.

### Clinical characteristics and outcomes

Clinical characteristics and outcomes were compared by cancer status (Table 2). More patients with active cancer (51.7%) were sourced from our institution’s emergency department, whereas more patients without cancer (53.9%) were transferred from an outside facility (*P* = 0.0002). Patients with active cancer had a greater number of ASMs and underlying medical comorbidities compared to patients without active cancer (*P* = 0.017 and *P* = 0.008, respectively). Many patients with active cancer who underwent emergent laparotomy did not have documented preoperative localization of their abdominal pathology to a specific anatomic region (e.g., foregut, small bowel, large bowel, nonenteric organ) compared to patients without cancer (46.1% *versus* 25.3%, *P* = 0.0001). Body mass index was not different between patients with or without active cancer. Patients with active cancer were not discharged home to care for themselves independently as frequently as patients without cancer (25.7% *versus* 48.5% discharged home, *P* = 0.0001). Patients with active cancer had higher rates of index mortality (16.9% *versus* 9.1%, *P* = 0.0359), 90-d systemic complications (29.7% *versus* 14.4%, *P* = 0.0020), and 90-d mortality (27.0% *versus* 3.1%, *P* = 0.0001) compared to patients without active cancer.

Univariate and multivariable models were completed for index systemic complications, index mortality, 90-d systemic complications, and 90-d mortality were calculated for patients with active cancer using patients without cancer as reference (Fig.). After adjusting for age, sex, race, ASMs, and Elixhauser comorbidities, patients with cancer had 2.4 times the odds of developing systemic complications within 90 d of discharge (aOR = 2.4, 95% confidence interval [CI]: [1.26, 4.50]) and 12.0 times the odds of dying within 90 d of discharge (aOR = 12.0, 95% CI: [4.9, 29.3]). Adjusted odds of morality and systemic complications during hospitalization were not significant.

### Cancer-specific outcomes

Details on cancer diagnosis and management were collected for the 89 patients with cancer (Table 3). Of the patients with active cancer, 60.7% had stage IV disease and were undergoing active treatment. Chemotherapy was the most common treatment (53.9%), followed by radiation therapy (11.24%) and immunotherapy (3.4%). Of the 54 patients undergoing active therapy, 36 were on first-line therapy, and 32 had recent clinical notes indicating progression of disease. Finally, 30.3% of patients with active cancer underwent palliative care consultation during admission.

Of the 89 patients with active cancer, 15 (17%) died while inpatient (Table 4). Palliative care consultation was more common among patients who died while hospitalized compared to those who survived (53.3% *versus* 25.7%, *P* = 0.034). Inpatient mortality did not differ by cancer subtype, stage IV disease, active treatment approach, use of first-line therapy, or reported treatment response. Stage IV disease was more common among patients who died within 90 d of discharge compared to those who survived (80.0% *versus* 53.7%, *P* = 0.039). Mortality rates within 90 d did not differ by cancer subtype, active treatment approach, use of first-line therapy, reported treatment response, or palliative care consultation during admission.

Of the 89 patients with active cancer, 42 (47.2%) experienced a systemic complication during hospitalization (Table 5). Palliative care consultation was more common among patients who experienced a systemic complication (45.2% *versus* 17.0%, *P* = 0.004). Inpatient complication rates did not differ by cancer subtype, stage IV disease, active treatment technique, use of first-line therapy, or treatment response. Systemic complication rates within 90 d of discharge did not differ by cancer subtype, stage IV disease, active treatment technique, use of first-line therapy, treatment response, or palliative care consultation during admission.

For patients with active cancer, odds ratios were computed for mortality and systemic complications both during hospitalization and 90 d after discharge (Table 6). All odds ratios were adjusted for age, sex, race, ASMs, and Elixhauser comorbidities. Patients with hematologic malignancies had 7.1 times the odds of developing 90-d mortality compared to patients with any other cancer subtype (aOR = 7.1, 95% CI: [1.24, 40.76]). Cancer subtype, chemotherapy, radiation therapy, and use of first-line treatment did not affect the odds of in-hospital or 90-d mortality.

## Discussion

Several efforts have been made to better understand outcomes following surgical intervention among cancer patients, with few focusing specifically on outcomes among cancer patients with abdominal emergencies. In this study, patients with cancer who underwent emergent laparotomy experienced higher odds of mortality within 90 d of laparotomy compared to patients without cancer.

Patients with cancer also experienced a higher burden of systemic complications within 90 d of laparotomy compared to patients without cancer. These patients were discharged more frequently to hospice and less frequently independently to home. This supports our hypothesis that this group of patients fares worse than their counterparts following emergent laparotomy and requires additional resources to recover, such as physical therapy and social support. These data bring to light concerns regarding the negative impacts of aggressive treatments, such as major surgical intervention, given the life-limiting nature of emergency surgical disease and increased need for convalescent resources, such as home nursing care, within this cohort.

Another component of this study included identifying potential drivers of morbidity and mortality among patients with cancer following emergent laparotomy. Patients with cancer were more acutely and chronically ill on initial presentation, as evidenced by their high ASM and Elixhauser Comorbidity score. As expected, poor surgical outcomes among patients with disseminated cancer who present with serious conditions, such as renal failure and sepsis, have been observed in other studies.^6^ In addition, patients with hematologic malignancies experienced over seven times the odds of mortality within 90 d, which differs from other cancer subtypes. High mortality among patients with hematologic malignancies undergoing abdominal operations has previously been described and may be secondary to innate or treatment-related immunologic dysfunction effectuating poor wound healing.^10,11^ In addition, Mokart *et al.* reported that preoperative septic shock and need for dialysis were independent risk factors for hospital mortality among patients with hematologic diseases undergoing emergent laparotomy.^12^ Accordingly, patients with hematologic malignancies may require more targeted perioperative counseling, as well as prompt identification and treatment of disease-related risk factors.

Although there is no universal risk stratification tool for patients with cancer undergoing emergency abdominal surgery given the heterogeneity of malignancies and treatment regimens, other studies have found that National Surgical Quality Improvement Program comorbidity-based modified Frailty-Index 5 has been predictive of poor postoperative outcomes among patients with some malignancies.^13^ In addition, frailty defined by the Clinical Frailty Scale has been associated withpoorpostoperativeoutcomesamongEGSpatientsaged65 andolder.^14^ Given the high rates of frailty among patients with cancer,^15^ frailty status should be a factor considered by acute care surgeons during within the preoperative timeframe.

Furthermore, among patients with cancer, those with stage IV malignancies had significantly greater mortality within 90-d of laparotomy. Advanced-stage cancer has previously been linked to poor outcomes at 30-d postlaparotomy for acute abdominal pathology^6^; this poses the question of whether emergent laparotomies among this population are presented with treatment-directed or palliative intent, with the latter focusing on symptom management and quality of life. Although some palliative procedures in patients with advanced-stage cancer have been shown to improve symptom burden,^16^ there is less certainty regarding treatment goals among these patients undergoing emergent operative procedures. Although our study did not focus on quality-of-life assessments, the increased morbidity and mortality in patients with advanced-stage cancer suggests these quality of life conversations are incredibly important to have in this patient population. For patients whose life expectancy is already so limited, a large emergent operation leaving them with decreased functionality may be doing more harm than benefit.

The role of palliative care among patients with cancer presenting with acute surgical disease is critical. In this study, approximately a third of patients with cancer underwent palliative care consultation during their hospitalization. In addition, patients with cancer who experienced systemic complications or mortality during their hospitalization more often underwent palliative care consultation compared to their counterparts. Although it is unclear whether these consultations were occurring before or following surgery, previous studies have demonstrated that surgical patients who necessitate palliative care typically do not undergo consultation until the final 24–48 h of life.^17–19^ Given the high morbidity and mortality of emergency surgeries near end-of-life in conjunction with the benefits of palliative care, such as improved advanced care planning,^20^ palliative care teams should be involved before emergent surgical intervention among patients with cancer. Cauley *et al.* analyzed outcomes among 875 patients with cancer undergoing emergency abdominal surgery and found that relatively very few (4%) patients had a do not resuscitate order placed preoperatively, suggesting that goals-of-care conversations were not being held until after acute conditions arose.^6^ This finding aligns with those from other studies, which suggests that very few palliative care consultations are conducted before high-risk surgical interventions.^21^

Given that patients with cancer in this study were more ill on initial surgical consultation, clinical practices in which acute care surgeons collaborate with palliative care teams and patients before surgical intervention should be implemented. Often the availability of the palliative and oncologic teams can be a barrier to involving them in patient discussions in an emergent setting. One possible solution would be to involve palliative care in an outpatient setting for patients identified as high risk of experiencing abdominal catastrophe so that the patients’ goals of care are well-established in case of emergency. Although oncologists may not have surgical expertise, they are qualified to identify patients at high risk of adverse outcomes. This would include patients with widely metastatic or peritoneal disease, those receiving aggressive oncologic therapies, or those with significantly limiting pre-existing conditions or malnutrition. Involving palliative care at oncology appointments could help set base-line goals of care regarding quality of life and what measures a patient would be willing to undergo in case of emergency. Even if palliative and oncology cannot be consulted before surgery, our study supports involving them as early in the admission as possible. This could help avoid additional operative interventions from occurring if the expected outcome is futile or not in line with the patient’s goals for quality of life.

There were various limitations in this study. Data were collected from a single institution and our sample size was limited, hindering both the statistical power of the study and generalizability. Given the retrospective nature of the study, certain laboratory values were unable to be accounted for if not present in the patients’ chart. We were not able to assess differences in outcomes among specific oncologic therapies given the heterogeneity of treatments in our sample size. In addition we were not able to specifically comment on patients in the survivorship phase. Expanding institution number and sample size would increase the power of the study, as a larger patient population may show more differences. In addition, patients who were diagnosed with cancer intraoperatively and during index hospitalization were not captured, potentially limiting the size of the cancer group and augmenting the collected cancer characteristics.

Patients with cancer have high odds of mortality and systemic complications within 90 d of undergoing emergency laparotomy. Early involvement of palliative care is crucial among these patients to provide a framework for advanced care planning. Understanding factors associated with poor outcomes among this subset of patients is crucial during preoperative assessment and risk counseling.

Larger-scale investigation is required to understand drivers of poor outcomes among patients with cancer undergoing emergent laparotomy. In doing this, risk stratification tools may be developed that can help guide perioperative decision-making. Given the high morbidity and mortality of emergency laparotomy among patients with cancer, it is critical that providers establish goals of care with patients early during the perioperative period. In addition, prospective research evaluating the impact of targeted perioperative counseling with early palliative care involvement may be helpful in minimizing poor outcomes among this patient population.

## Figures and Tables

**Fig. –:**
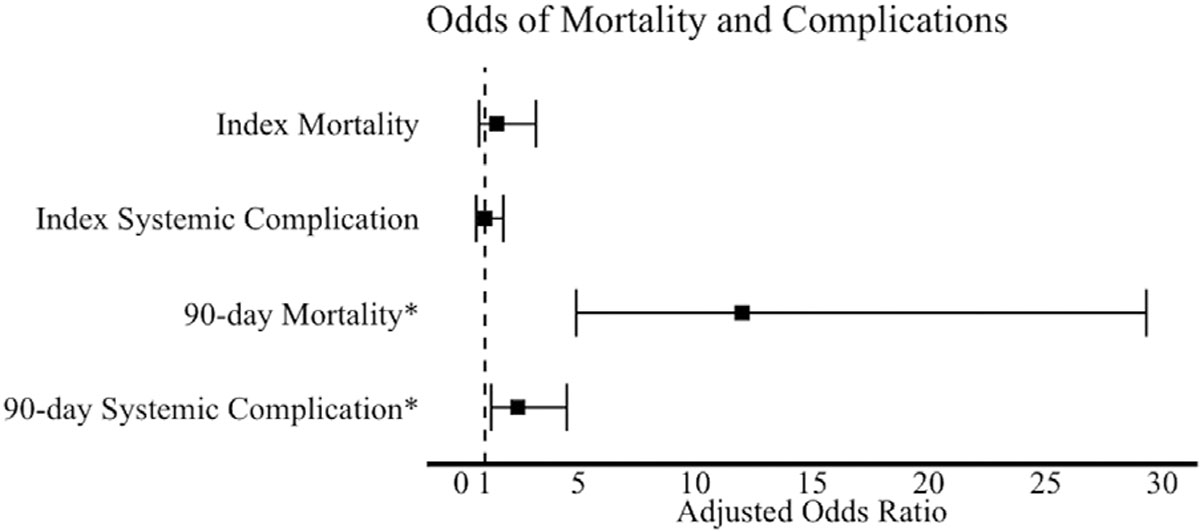
Odds ratios are shown for mortality and systemic complications during hospitalization (index) and 90 d after discharge (90-d) for patients with active cancer using patients without cancer as reference. All odds ratios were adjusted for age, sex, race, acute severity measures, and Elixhauser comorbidities. Squares indicate adjusted odds ratios. Whiskers indicate the 95% confidence interval range. A dotted line is shown at an adjusted odds ratio of one. Significance is indicated with an asterisk and defined as P< 0.05.

**Table 1 –:** Patient demographics

Variable	No cancer (*N* = 320)	Active cancer (*N* = 89)	*P* value

Sex, female	167 (51.8)	37 (41.2)	0.0765
Median age in years (IQR)	59 [49, 69]	63 [56, 70]	0.0001
Age			0.0018*
18–24	9 (2.8)	1 (1.1)	
25–44	55 (17.2)	3 (3.4)	
45–64	141 (44.1)	43 (48.3)	
65–84	100 (31.3)	41 (46.1)	
85+	15 (4.7)	1 (1.1)	
Ethnicity			0.2354
Hispanic	5 (1.6)	0 (0.0)	
Race			0.6958
White	260 (81.3)	74 (83.2)	
Black	41 (12.8)	12 (13.5)	
Other	15 (4.7)	3 (3.4)	
Unknown	4 (1.3)	0 (0.0)	
Insurance			0.1246
Private	83 (25.9)	21 (23.6)	
Medicare	141 (44.1)	51 (57.3)	
Medicaid	57 (17.8)	10 (11.2)	
Other	39 (12.2)	7 (7.9)	
Employment status			0.2527
Unemployed	90 (28.1)	23 (25.8)	
Employed	67 (20.9)	21 (23.6)	
Retired	71 (22.2)	28 (31.5)	
Disabled	50 (15.6)	10 (11.2)	
Unknown	42 (13.1)	7 (7.9)	
Lives independently	55 (17.2)	18 (20.2)	0.5081
Quality of social support			0.0609
Unsupportive	18 (5.6)	6 (6.7)	
Supportive	225 (70.3)	56 (62.9)	
Very supportive	38 (11.9)	20 (22.5)	
Unknown/missing	39 (12.2)	7 (7.9)	

Continuous variables are represented as median [IQR]. Categorical variables are represented as count (percentage).

* Significant at *P*-value .05.

**Table 2 –:** Clinical characteristics and outcomes.

Variable	No cancer (*N* = 320)	Active cancer (*N* = 89)	*P* value

ED source			0.0002*
Home	133 (42.9)	45 (51.7)	
Transfer from facility	167 (53.9)	31 (35.6)	
Other	10 (3.2)	11 (12.6)	
Acute severity measures			0.0167*
0	165 (51.6)	30 (33.7)	
1	76 (23.8)	25 (28.1)	
2	37 (11.6)	18 (20.2)	
3 or more	42 (13.1)	16 (17.9)	
Elixhauser comorbidities			0.0081*
0	35 (10.9)	0 (0.0)	
1	41 (12.8)	10 (11.2)	
2	67 (20.9)	25 (28.1)	
3 or more	177 (55.3)	54 (60.7)	
Preoperative diagnosis			0.0001*
Foregut	14 (4.4)	3 (3.4)	
Small bowel	155 (48.4)	21 (23.6)	
Large bowel	51 (15.9)	23 (25.8)	
Nonenteric organ	19 (5.9)	1 (1.1)	
Unable to determine	81 (25.3)	41 (46.1)	
BMI category (*n*, %)			0.2997
Underweight <18.5	15 (4.7)	5 (5.6)	
Normal weight 18.5–24.9	91 (28.6)	33 (37.1)	
Overweight 25–29.9	83 (26.1)	24 (27.0)	
Obesity ≥ 30	129 (40.6)	27 (30.3)	
Outcomes			
Discharge disposition			0.0001*
Home	141 (48.5)	19 (25.7)	
Home with services	60 (20.6)	24 (32.4)	
Care facility	81 (27.8)	22 (29.7)	
Hospice	0 (0.0)	8 (10.8)	
Other/unknown	9 (3.1)	1 (1.4)	
Palliative consult			0.0001*
Yes	16 (5.0)	26 (29.2)	
Index systemic complication	122 (38.1)	42 (47.2)	0.1227
Index mortality	21 (9.1)	15 (16.9)	0.0359*
90-d systemic complications	42 (14.4)	22 (29.7)	0.0020*
90-d mortality	9 (3.1)	20 (27.0)	0.0001*

BMI, body mass index.

* Significant at *P* value 0.05.

**Table 3 –:** Cancer characteristics.

Variable	*N* = 89 (%)

Cancer subtype	
Foregut	6 (6.7)
Colorectal/anal	26 (29.2)
Hepatopancreatobiliary	6 (6.7)
Breast/skin/soft tissue	9 (10.1)
Blood	14 (15.7)
Other	28 (31.5)
Stage IV disease	54 (60.7)
Active treatment*	54 (60.7)
Chemotherapy	48 (53.9)
Radiation	10 (11.24)
Immunotherapy	3 (3.4)
Hormonal therapy	0 (0.0)
First-line therapy	36 (66.7)
Treatment response	
Progression of disease	32 (36.0)
Response to treatment	3 (3.4)
Stable	6 (6.7)
Unknown	13 (14.6)
Palliative care consultation during admission	27 (30.3)

Continuous variables are represented as median [IQR]. Categorical variables are represented as count (percentage).

* Active treatment defined as within 30 d before presentation.

**Table 4 –:** Mortality in cancer patients.

Variable	Inpatient			90-D^†^		
	No *n* (%) *N* = 74	Yes *n* (%) *N* = 15	*P* value	No *N* = 54 (%)	Yes *N* = 20 (%)	*P* value

Cancer subtype			0.449			0.576
Foregut	5 (6.8)	1 (6.7)		4 (7.4)	1 (5.0)	
Colorectal/anal	21 (28.4)	6 (33.3)		13 (24.1)	8 (40.0)	
Hepatopancreatobiliary	4 (5.4)	2 (13.3)		4 (7.4)	0 (0.0)	
Breast/skin/oft tissue	8 (10.8)	1 (6.7)		5 (9.3)	3 (15.0)	
Blood	10 (13.5)	4 (26.7)		8 (14.8)	2 (10.0)	
Other	26 (35.1)	2 (13.3)		20 (37.0)	6 (30.0)	
Stage IV disease	45 (60.8)	9 (60.0)	0.953	29 (53.7)	16 (80.0)	0.039*
Active treatment‡	43 (58.1)	11 (73.3)	0.270	29 (53.7)	14 (70.0)	0.207
Chemotherapy	38 (51.4)	10 (66.7)	0.278	26 (48.2)	12 (60.0)	0.365
Radiation	9 (12.2)	1 (6.67)	0.539	6 (11.1)	3 (15.0)	0.649
Immunotherapy	3 (4.1)	0 (0.0)	0.428	2 (3.7)	1 (5.0)	0.802
First-line therapy	29 (39.2)	7 (46.7)	0.591	18 (33.3)	11 (55.0)	0.090
Treatment response						
Progression of disease	27 (36.5)	5 (33.3)	0.817	17 (31.5)	10 (50.0)	0.142
Response to treatment	3 (4.1)	0 (0.0)	0.428	3 (5.6)	0 (0.0)	0.282
Stable	5 (6.8)	1 (6.7)	0.990	3 (5.6)	2 (10.0)	0.499
Unknown	8 (10.8)	5 (33.3)	0.024*	6 (11.1)	2 (10.0)	0.891
Palliative care consultation during admission	19 (25.7)	8 (53.3)	0.034*	11 (20.4)	8 (40.0)	0.086

Categorical variables are represented as count (percentage).

* Significant at *P* value 0.05.

† Only includes those discharged alive.

‡ Active treatment defined as within 30 d before presentation.

**Table 5 –:** Systemic complications in cancer patients.

Variable	Inpatient			90-D		
	No *n* (%) *N* = 47	Yes *n* (%) *N* = 42	*P*-value	No *n* (%)	Yes *n* (%)	*P* value

Cancer subtype			0.636			0.656
Foregut	2 (4.3)	4 (9.5)		4 (7.7)	1 (4.6)	
Colorectal/anal	16 (34.0)	10 (23.8)		17 (32.7)	4 (18.2)	
Hepatopancreatobiliary	2 (4.3)	4 (9.5)		3 (5.8)	1 (4.6)	
Breast/skin/soft tissue	5 (10.6)	4 (9.5)		4 (7.7)	4 (18.2)	
Blood	6 (12.8)	8 (19.1)		7 (13.5)	3 (13.6)	
Other	16 (34.0)	12 (28.6)		17 (32.7)	9 (40.9)	
Stage IV disease	25 (53.2)	29 (69.1)	0.126	31 (59.6)	14 (63.6)	0.746
Active treatment^†^	28 (59.6)	26 (61.9)	0.822	32 (61.5)	11 (50.0)	0.358
Chemotherapy	25 (53.2)	23 (54.8)	0.882	28 (53.9)	10 (45.5)	0.509
Radiation	6 (12.8)	4 (9.5)		6 (11.5)	3 (13.6)	0.801
Immunotherapy	2 (4.3)	1 (2.4)		3 (5.8)	0 (0.0)	0.250
First-line therapy	19 (40.4)	17 (40.5)	0.996	22 (42.3)	7 (31.8)	0.398
Treatment response						
Progression of disease	15 (31.9)	17 (40.5)	0.401	21 (40.4)	6 (27.3)	0.284
Response to treatment	2 (4.3)	1 (2.4)	0.625	3 (5.8)	0 (0.0)	0.250
Stable	5 (10.6)	1 (2.4)	0.121	4 (7.7)	1 (4.6)	0.622
Unknown	6 (12.8)	7 (16.7)	0.603	4 (7.7)	4 (18.2)	0.184
Palliative care consultation during admission	8 (17.0)	19 (45.2)	0.004*	13 (25.0)	6 (27.3)	0.838

Categorical variables are represented as count (percentage).

* Significant at *P* value 0.05.

† Active treatment defined as within 30 d before presentation.

**Table 6 –:** Adjusted* odds ratios for systemic complications and mortality.

Variable	Inpatient			90-D		
	aOR	95% LCL	95% UCL	aOR	95% LCL	95% UCL

Mortality						
Cancer subtype						
Foregut	0.8	0.04	15.16	1.4	0.07	28.83
Colorectal/anal	1.2	0.19	7.04	5.4	0.95	30.74
Hepatopancreatobiliary	1.6	0.14	18.04	-	-	-
Breast/skin/soft tissue	0.3	0.02	6.69	3.2	0.31	32.93
Blood	2.2	0.54	8.42	7.1^†^	1.24	40.76
Other	0.3	0.04	2.93	2.3	0.34	16.10
Clinical management						
Chemotherapy	1.7	0.57	5.1	1.6	0.48	5.37
Radiation	0.5	0.05	4.81	1.3	0.24	7.32
On first-line treatment	1.3	0.42	4.21	2.9	0.94	9.19
Systemic complication						
Cancer subtype						
Foregut	0.7	0.08	6.64	1.3	0.09	18.66
Colorectal/anal	0.3	0.09	1.22	1.0	0.22	4.59
Hepatopancreatobiliary	0.7	0.08	5.54	1.3	0.10	16.38
Breast/skin/soft tissue	0.2	0.03	1.43	4.6	0.68	30.46
Blood	1.3	0.40	4.54	2.2	0.51	9.40
Other	0.4	0.12	1.48	2.7	0.73	9.67
Clinical management						
Chemotherapy	0.6	0.26	1.53	0.8	0.26	2.35
Radiation	0.5	0.12	2.13	1.4	0.29	6.37
On first-line treatment	0.7	0.29	1.76	0.7	0.21	2.07

aOR = adjusted odds ratio; LCL = lower confidence level; UCL = upper confidence level.

* Adjusted for age, sex, race, Elixhauser comorbidities, acute severity measures, and disseminated cancer.

† Significant at 5% significance level.

## References

[1] Havens JM, Peetz AB, Do WS, The excess morbidity and mortality of emergency general surgery. J Trauma Acute Care Surg. 2015;78:306–311. 25757115 10.1097/TA.0000000000000517

[2] Ilgen JS, Marr AL. Cancer emergencies: the acute abdomen. Emerg Med Clin North Am. 2009;27:381–399. 19646643 10.1016/j.emc.2009.04.006

[3] Guimaraes MD, Bitencourt AG, Marchiori E, Chojniak R, Gross JL, Kundra V. Imaging acute complications in cancer patients: what should be evaluated in the emergency setting? Cancer Imaging. 2014;14:18. 25609051 10.1186/1470-7330-14-18 PMC4331823

[4] Bosscher MRF, Van Leeuwen BL, Hoekstra HJ. Surgical emergencies in oncology. Cancer Treat Rev. 2014;40:1028–1036. 24933674 10.1016/j.ctrv.2014.05.005

[5] Leeds IL, Canner JK, Efron JE, The independent effect of cancer on outcomes: a potential limitation of surgical risk prediction. J Surg Res. 2017;220:402–409.e6. 28923559 10.1016/j.jss.2017.08.039 PMC5712450

[6] Kassahun WT, Babel J, Mehdorn M. Assessing differences in surgical outcomes following emergency abdominal exploration for complications of elective surgery and high-risk primary emergencies. Sci Rep. 2022;12:1349. 35079087 10.1038/s41598-022-05326-4 PMC8789789

[7] Cauley CE, Panizales MT, Reznor G, Outcomes after emergency abdominal surgery in patients with advanced cancer: opportunities to reduce complications and improve palliative care. J Trauma Acute Care Surg. 2015;79:399–406. 26307872 10.1097/TA.0000000000000764 PMC4552078

[8] Harris PA, Taylor R, Minor BL, The REDCap consortium: building an international community of software platform partners. J Biomed Inform. 2019;95:103208. 31078660 10.1016/j.jbi.2019.103208 PMC7254481

[9] Harris PA, Taylor R, Thielke R, Payne J, Gonzalez N, Conde JG. Research electronic data capture (REDCap)—a metadata-driven methodology and workflow process for providing translational research informatics support. J Biomed Inform. 2009;42:377–381. 18929686 10.1016/j.jbi.2008.08.010 PMC2700030

[10] Forrester JD, Syed M, Tennakoon L, Spain DA, Knowlton LM. Mortality after general surgery among hospitalized patients with hematologic malignancy. J Surg Res. 2020;256:502–511. 32798998 10.1016/j.jss.2020.07.006

[11] Von Kroge PH, Duprée A, Mann O, Abdominal emergency surgery in patients with hematological malignancies: a retrospective single-center analysis. World J Emerg Surg. 2023;18:12. 36747231 10.1186/s13017-023-00481-z PMC9900956

[12] Mokart D, Penalver M, Chow-Chine L, Surgical treatment of acute abdominal complications in hematology patients: outcomes and prognostic factors. Leuk Lymphoma. 2017;58:2395–2402. 28278710 10.1080/10428194.2017.1296145

[13] Chambers LM, Chalif J, Yao M, Modified frailty index predicts postoperative complications in women with gynecologic cancer undergoing cytoreductive surgery and hyperthermic intraperitoneal chemotherapy. Gynecol Oncol. 2021;162:368–374. 34083027 10.1016/j.ygyno.2021.05.013

[14] Fehlmann CA, Patel D, McCallum J, Perry JJ, Eagles D. Association between mortality and frailty in emergency general surgery: a systematic review and meta-analysis. Eur J Trauma Emerg Surg. 2022;48:141–151. 33423069 10.1007/s00068-020-01578-9 PMC8825621

[15] Resendes NM, Oomrigar SM, Tannous T, Ruiz JG, Hammel IM. The association of cancer diagnosis with frailty status in high need, high risk veterans. J Clin Oncol. 2022;40:e24024.

[16] Deo SVS, Kumar N, Rajendra VKJ, Palliative surgery for advanced cancer: clinical profile, spectrum of surgery and outcomes from a tertiary care cancer centre in low-middle-income country. Indian J Palliat Care. 2021;27:281–285. 34511797 10.25259/IJPC_399_20 PMC8428898

[17] Olmsted CL, Johnson AM, Kaboli P, Cullen J, Vaughan-Sarrazin MS. Use of palliative care and hospice among surgical and medical specialties in the veterans Health administration. JAMA Surg. 2014;149:1169. 25251601 10.1001/jamasurg.2014.2101

[18] Rodriguez R, Marr L, Rajput A, Fahy BN. Utilization of palliative care consultation service by surgical services. Ann Palliat Med. 2015;4:194–199. 26541398 10.3978/j.issn.2224-5820.2015.09.03

[19] Wilson DG, Harris SK, Peck H, Patterns of care in hospitalized vascular surgery patients at end of life. JAMA Surg. 2017;152:183. 27806150 10.1001/jamasurg.2016.3970

[20] Cooper Z, Corso K, Bernacki R, Bader A, Gawande A, Block S. Conversations about treatment preferences before high-risk surgery: a pilot study in the preoperative testing center. J Palliat Med. 2014;17:701–707. 24832687 10.1089/jpm.2013.0311

[21] Yefimova M, Aslakson RA, Yang L, Palliative care and end-of-life outcomes following high-risk surgery. JAMA Surg. 2020;155:138. 31895424 10.1001/jamasurg.2019.5083 PMC6990868

